# The common drivers of children and young people’s health and wellbeing across 13 local government areas: a systems view

**DOI:** 10.1186/s12889-024-18354-8

**Published:** 2024-03-19

**Authors:** Siobhan A. O’Halloran, Joshua Hayward, Melissa Valdivia Cabrera, Tiana Felmingham, Penny Fraser, Cindy Needham, Jaimie Poorter, Doug Creighton, Michael Johnstone, Melanie Nichols, Steven Allender

**Affiliations:** 1 Global Centre for Preventive Health and Nutrition, Institute for Health Transformation, School of Health and Social Development, Faculty of Health, Deakin University, Geelong, VIC 3220 Australia; 2 Institute for Intelligent Systems Research and Innovation, Deakin University, 75 Pigdons Rd, Waurn Ponds, VIC 3216 Australia

**Keywords:** Social network analysis, Local government, Systems thinking, Community, Health

## Abstract

**Background:**

System dynamics approaches, including group model building (GMB) and causal loop diagrams (CLDs), can be used to document complex public health problems from a community perspective. This paper aims to apply Social Network Analysis (SNA) methods to combine multiple CLDs created by local communities into a summary CLD, to identify common drivers of the health and wellbeing of children and young people.

**Methods:**

Thirteen community CLDs regarding children and young people health and wellbeing were merged into one diagram involving three steps: (1) combining variable names; (2) CLD merging, where multiple CLDs were combined into one CLD with a set of unique variables and connections; (3) paring, where the Decision-Making Trial and Evaluation Laboratory (DEMATEL) method was used to generate a cut-point to reduce the number of variables and connections and to rank the overall importance of each variable in the merged CLD.

**Results:**

Combining variable names resulted in 290 variables across the 13 CLDS. A total of 1,042 causal links were identified in the merged CLD. The DEMATEL analysis of the merged CLD identified 23 common variables with a net importance between 1.0 and 4.5 R + C values and 57 causal links. The variables with the highest net importance were ‘mental health’ and ‘social connection & support’ classified as high net receivers of influence within the system.

**Conclusions:**

Combining large CLDs into a simple diagram represents a generalisable model of the drivers of complex health problems.

## Background

Lifestyle related chronic diseases such as obesity, diabetes, cancer and cardiovascular disease are influenced by multiple modifiable risk factors. The complex range and interactions of these risks and their determinants make it difficult to understand and address layers of cause and effect [1]. Methods and practises from system dynamics are becoming more commonly used to understand and address the complexity of health issues [2, 3]. Applicable methods include group model building (GMB), where stakeholders are engaged to capture complexity in graphic representations like causal loop diagrams (CLDs) [4]. CLDs are comprised of variables that are dynamic causes or effects of the complex problem, and arrows that represent hypothesised causal relationships between the variables [4]. The orientation of each relationship is either positive, where variables change in the same direction, or negative, where variables change in the opposite direction (known as positive or negative polarity) [5]. Both methods are increasingly being applied in population health research as they provide tangible, participatory methods to explore systems thinking concepts such as non-linearity, unintended consequences and time delays in systems [6]. The growing focus on using these techniques in co-design of community-based prevention approaches is based on the hypothesis that enhanced understanding of these systems by community stakeholders is required to ensure good design and successful implementation [7]. 

Promising examples of using co-design as an approach to address complexity in public health issues are beginning to emerge in the literature. One recent study demonstrated GMB processes with collaborative, cross-sectoral participant groups, [8] while others have described whole-of-community systems-based prevention initiatives within Australia [9–11] and internationally [12, 13]. As examples of these projects continue to grow, attention has turned to understanding and comparing how multiple communities attempt to recognise the commonalities of chronic disease risk (e.g., poor nutrition, lack of physical activity). Determining these commonalities may be valuable for identifying key population-level drivers and potential solutions. CLDs have been used with large numbers of locally based government and community members and are a unique representation of the stakeholder perspectives in a community. More recently multiple CLDs have been generated across a number of communities within the same project, targeting the same outcome [10]. An example is the VicHealth Local Government Partnership working in multiple municipalities, building CLDs independently from each other as part of public health planning; and the findings, from each CLD providing additional insight for other municipalities that were not revealed during their independent GMB workshops [10]. Another example is the ‘Confronting obesity: Co-creating policy with youth’ (CO-CREATE), which used a complex systems framework to explore the drivers of adolescent obesity and potential policy actions across five European countries [14]. Testing methods to combine and compare CLDs developed by multiple stakeholders is an important next step in the field to understand the factors that influence complex health problems at the population level.

Social Network Analysis (SNA), which involves investigating social structure and how it influences health through the use of networks and graph theory, has previously been used as a computational tool to analyse and combine CLDs [15]. One recent example was the use of SNA to converge 13 CLDs, from within one community, into a summary diagram designed to represent the common perspectives of the entire participant group [16]. The combined diagram was rated favourably by participants for its representation of their perspective, connection to other perspectives and insights and usefulness as a communication tool [16]. In this paper we extend these SNA methods to explore the utility for larger CLDs developed with multiple stakeholders and assess whether they can identify commonalities between different communities considering children and young people’s health and wellbeing from a systems perspective.

The aims of this paper were:

1. To apply SNA methods to combine the multiple CLDs created by local communities into one merged CLD containing the full set of unique variables and connections from each of the community diagrams.
2. To identify and describe the similar variables representing key drivers of the health and wellbeing of children and young people from the merged CLD.

## Methods

### Study context

The data used in this analysis were collected in GMB workshops facilitated by local government-based prevention workforce (referred to hereon as “councils”) as part of the VicHealth Local Government Partnership (VLGP), implemented across the state of Victoria, Australia. The VLGP has been described in detail in a prior publication [10]. Briefly, the partnership provides support to councils to deliver evidence-based actions to improve children and young people’s health and wellbeing. Eight evidence-informed health promotion modules were designed to provide a series of practical ‘how-to guides’ for policy, program development/delivery and practice change [10, 17]. One of the VLGP modules, Connecting the Dots (CtD), consisted of structured training and ongoing support for councils to deliver GMB sessions as a mechanism to incorporate systems thinking and community co-design principles into local government prevention practise [10, 17]. The resultant CLDs, created with community input from across each Local Government Authority, were used to inform relevant council health and wellbeing planning and action delivery until 2025.

### GMB workshops and CLD creation

The GMB workshops and CLD development has been described elsewhere [10, 17]. 

Thirteen councils completed the CtD module, and subsequently facilitated GMB workshops. The GMB process undertaken by councils was designed to be delivered across three interactive sessions, each 2–3 h in duration, although on occasions there were minor modifications to the process due to COVID19 restrictions and time constraints [18]. The GMB workshops engaged a range of participants including councils’ stakeholders and partnering organisations, and local community members (including children and young people) to develop a CLD that visually represented the interrelationships between the locally relevant drivers of the health and wellbeing of children and/or young people from the community’s perspective [10]. The systems mapping software program called Systems Thinking in Community Knowledge Exchange (STICKE) was used to build the CLDs during the workshops [10]. 

The workshops were structured around three GMB scripts: “Graphs over time,” (assists in framing a problem, to identify variables and gather input that influences the topic for the workshop process. It is used at the beginning of a workshop [18]); “Connection Circles,” (used after graphs over time, to identify connections between variables and additional variables not identified in graphs over time [18]); “Action Ideas” (used to identify and prioritize actions after a CLD has been developed [18]) which were adapted from the Scriptapedia database [19]. Each script was chosen to guide the facilitation of the workshop process and build the CLDs. These scripts have been applied in previous prevention trials [9, 11]. In the final workshop, council teams facilitated an action co-design process, using the CLD as a reference in order to identify intervention targets and corresponding community-led actions for consideration.

### Analysis

Developing commonality between the 13 CLDs comprised three steps; (1) Variable name combination (2) CLD merging, and (3) Paring (i.e., reducing the size of CLDs).

#### Variable name combination

All variable names across the 13 CLDs were reviewed and collapsed into unique combined variable names to ensure consistency in language across the data set [20]. Combination of variable names by commonality from the 13 CLDs was independently performed by two reviewers (SO’H and JH). Where variables clearly referred to the same construct but were named differently, a combined variable name that adequately reflected the construct was selected as a replacement. For example, the variables ‘consumption of healthy food’, ‘eating healthy food’, and ‘healthy eating’ were combined to ‘healthy food consumption’. In other instances, variables referring to different, but related constructs could be collapsed into a combined variable name. For example, the variable names, ‘substance abuse’, ‘alcohol and drug use’ ‘tobacco’, were collapsed together into a new variable called ‘alcohol and other substance use’. Following independent review and combination of the variable names, differences in the variables identified for combination, and final names selected, were reviewed and discussed between the two researchers, and a final decision was reached by consensus.

### CLD merging

Following variable combination, the 13 constituent CLDs were combined into a single CLD that contained the full set of unique variables and connections from the constituent diagrams. This was achieved by converting each diagram into an adjacency matrix, wherein relationships between each pair of diagram variables could be represented as a 0 (no connection), 1 (connection, positive relationship), or -1 (connection, negative relationship).

The matrices were then merged, with each unique variable name appearing only once, and connections appearing in more than one constituent diagram represented by increasing the integer used to signify a connection (i.e., where a positive connection existed in two constituent diagrams, the relevant cell in the merged matrix contained a “2”, etc.). Conflicting links were identified before the merge and the original maps were adjusted to resolve the conflicts by the map builders. Once all constituent CLD’s had been combined into the merged matrix, the matrix was converted back into a CLD by using STICKE.

### Paring

Finally, two cut points were used to reduce the merged CLD from the full set of variables names and connections present across all constituent CLD’s, to a summary diagram describing the key commonality between the 13 CLD’s.

Decision making trial and evaluation laboratory (DEMATEL) methods were used to generate a cut point that could be used to reduce the number of variables in the merged CLD [16]. DEMATEL is an analytical technique from SNA, used to rank components within networks by their influence, as determined by their position within the overall network structure [16]. The approach provides ranking metrics on the overall “importance” of each variable within a network, which can be conceptualised as the sum of its score as a net influencer and/or net receiver of influence [21]. Inspection of a scatterplot of the overall importance of diagram variables against their net “influencer” or “receiver of influence” score can identify clustering within the data, which was used in this case to generate and apply a cut-point to reduce the number of variables and connections in the merged CLD. This resulted in a sub-set of variables from the merged CLD, representing the most significant drivers of the health and wellbeing of children and young people, according to the aggregated perspective of participants from across the 13 constituent communities.

Notably, use of the DEMATEL approach necessitated the “loss” of connection polarity as the analysis requires all values relating to connections to be positive (i.e., when one variable increases, it causes an increase in another). However, the final diagram was reviewed, and polarity reintroduced with reference to the original data.

Lastly – after the final set of variables had been identified, a cut-point was applied to reduce the total number of connections in the diagram. While DEMATEL provides diagram structure it does not provide insight into where a cut point should be applied and so the research team set a cut point. To further reduce connections between variables, connections that appeared in only one of the original constituent diagrams (i.e., had a value of 1 or -1) were removed. This approach was intended to remove connections that had been identified by one community but were not confirmed as being relevant to other communities by appearing in at least one other diagram.

Of note, we did not observe a significant change to our final model when the cut-point was modified by 5,10 and 15% during a sensitivity analysis. For example, our initial cut-off point was of net importance 1.0. Increasing it by 5% excluded the variable “sleep” (net importance 1.02) and decreasing it by 5% did not include more variables. An increase of 10% excluded the variables “sleep” and “alcohol and other substance use” (net importance 1.09) without the inclusion of more variables. A 15% increase and decease excluded “sleep” and “alcohol and other substance use” and included “cultural awareness & safety” (net importance 0.89) and “availability of healthy food” (net importance 0.86) respectively.

## Results

Step 1 resulted in 290 unique variables across the 13 CLD’s. The collapsing of the 13 diagrams into one merged diagram (step 2) resulted in a total of 1,042 causal links. In step 3, DEMATEL analysis identified 23 variables with a net importance between 1.0 and 4.5 R + C (267 variables were excluded resulting in the final 23).The cut-point was selected based on the clear boundary between the two nodes on the scatter plot. DEMATEL provides an importance ranking and a balance is required to keep the most important variables, without resulting in an excessive number where the map becomes too visually complex.

**Fig. 1:**
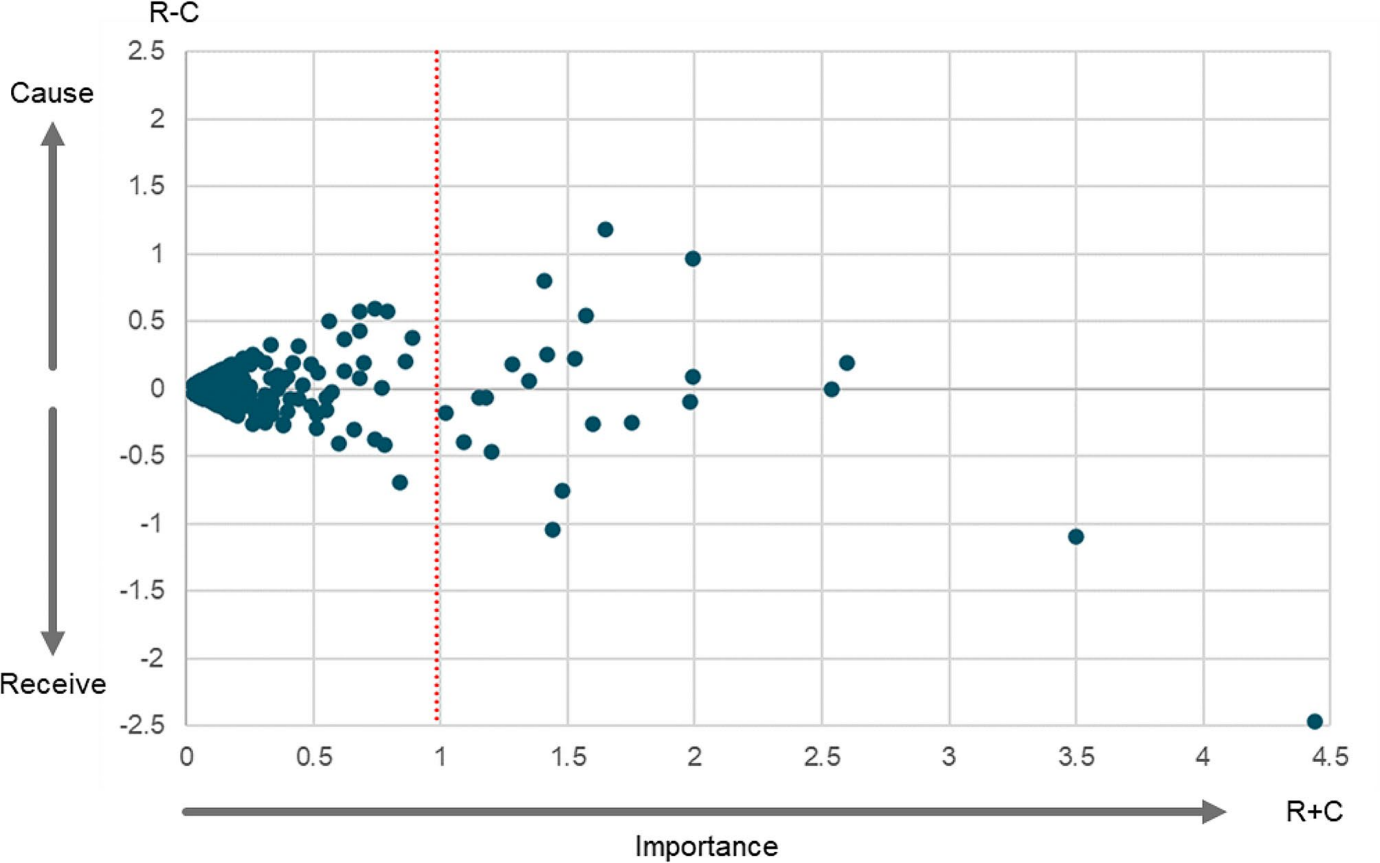
DEMATEL analysis indicating 23 variable cut point ranging from a net importance between 1.0 and 4.5 R + C value

Figure 1 shows the variables on a scatter plot, classified on the horizontal axis according to the total net importance (an output of the DEMATEL analysis, unrelated to the frequency of the variable appearing in the original maps) and on the vertical axis classified as positive if the variables were an overall network influencer (C) or as negative if the variable was a receiver of overall network influence (R).

Table 1 shows the 23 most important variables according to the DEMATEL R + C value across the 13 CLD’s. The variables with the highest net importance were ‘mental health’ and ‘social connection & support’ classified as high net receivers of influence within the system. The highest net influencers of the network were ‘access to transport’ and ‘financial security’.

**Table 1:** Variable rankings by DEMATEL score, and constituent CLD frequency

		DEMATEL		
	**Variable**	R + C	R-C	Frequency*
1	Mental health	4.44	-2.47	10
2	Social connection & support	3.5	-1.09	12
3	Access to services and resources	2.6	0.19	10
4	Sport and recreation	2.54	0	12
5	Financial security	1.99	0.97	10
6	Education and training	1.99	0.09	11
7	School attendance	1.98	-0.1	8
8	Work & employment opportunities	1.75	-0.25	9
9	Access to transport	1.65	1.18	9
10	Physical activity	1.6	-0.26	6
11	Healthy role modelling	1.57	0.54	8
12	Supportive family environment	1.53	0.22	9
13	Healthy food consumption	1.48	-0.75	7
14	Physical health and fitness	1.44	-1.04	4
15	Family violence	1.42	0.25	5
16	Social media	1.41	0.8	8
17	Bullying	1.35	0.06	6
18	Access to housing	1.28	0.18	11
19	Social isolation	1.2	-0.47	3
20	Activities and events	1.18	-0.06	9
21	Safe and inclusive environments	1.15	-0.06	5
22	Alcohol and other substance use	1.09	-0.39	5
23	Sleep	1.02	-0.18	6

*Frequency is the occurrence in the original maps

**Fig. 2:**
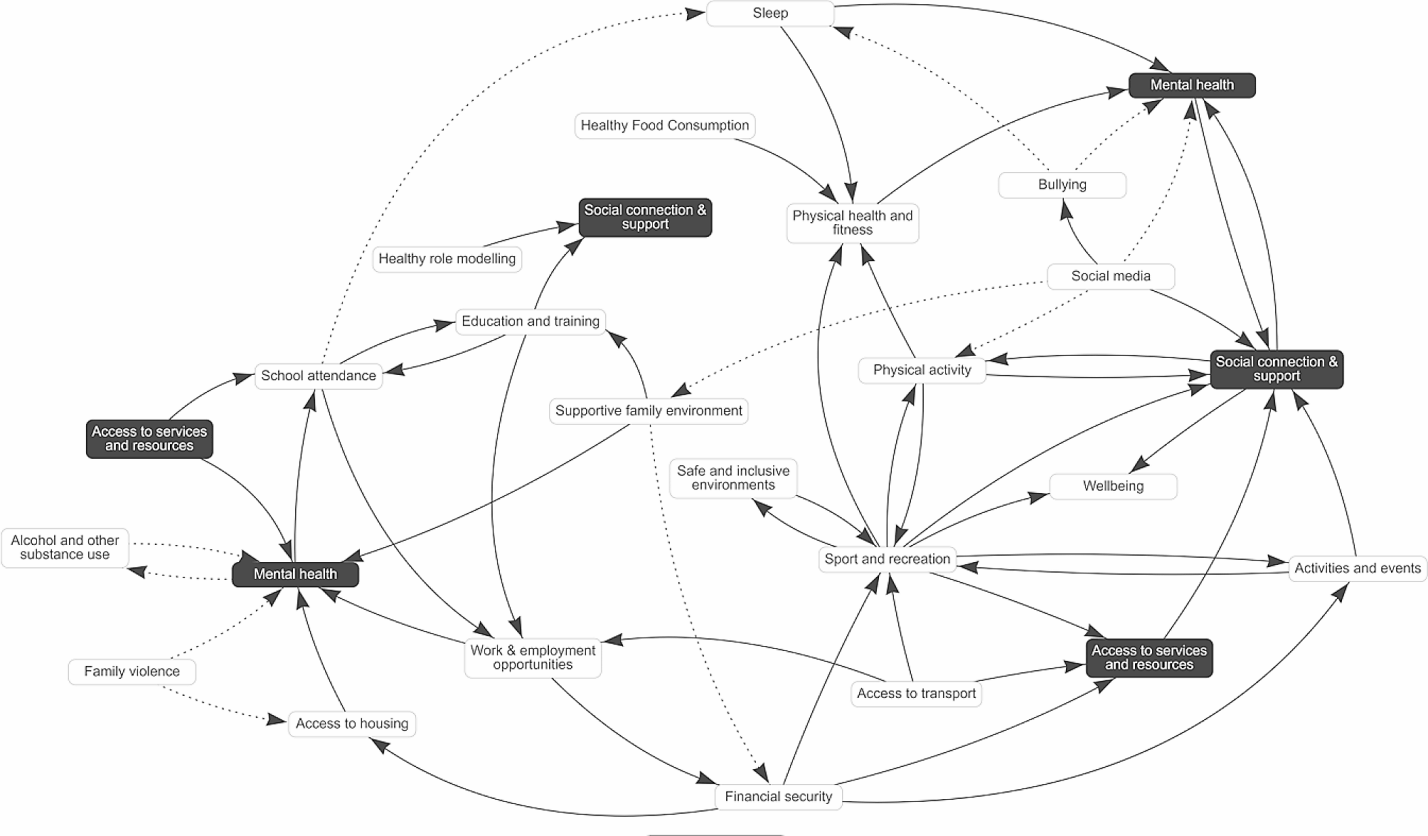
Final merged CLD after applying DEMATEL and causal connection cut points. Positive causal relationships are denoted by a solid arrow and negative causal relationships by a dashed arrow. Variables marked in black were duplicated in several places on the CLD to minimize arrow crossover and improve readability

The variables were also analysed according to their frequency of occurrence across the 13 constituent diagrams. ‘Social connection & support’ and ‘sport and recreation’ had the highest frequency, appearing in 12 out of the 13 CLDs, while ‘social isolation’ appeared fewest times in 3. Using the DEMATEL cut point, the merged diagram retained 155 causal connections. Application of the 2-CLD cut point to connections resulted in a final set of 57 causal connections. Figure 2 shows the final CLD with the most important variables and their connections representing the most important drivers of the health and wellbeing of children and young people.

Data including a list of the excluded variables can be obtained by contacting the corresponding author.

## Discussion

This study presents a method for collating causal loop diagrams across communities where comparable group model building processes have been employed to understand the common drivers of children and young people’s health and wellbeing in 13 communities. The method for combining these ‘system maps’ was able to condense a very large number of variables and connections to a reduced number of related terms and mathematically derive a summary model of the most critical variables common across all communities. The ability to objectively collate and describe data from GMBs across 13 communities represents an important step to creating generic models of the key commonalities of complex health issues such as causes of chronic disease, that includes stakeholder’s perspectives.

As the use of system methods in co-creation continues to grow, new analytic techniques such as those presented here will be needed to gather full insight across multiple communities. There are few previous examples, a notable exception being the work of Brennan and colleagues which used similar steps to reduce the size of the variable list across CLDs about healthy eating, active living and child obesity from 49 different communities [22]. The resulting CLD was larger than that presented here, including 227 unique variables coded into five thematic areas. Their findings about key determinants (healthy eating policies and environments, active living policies and environments, health and health behaviors, partnership and community capacity, and social determinants) parallel several of our findings that key drivers include social connection and support, supporting environments and access to services.

A characteristic of systems methods when used to address complex public health issues is the focus on understanding multiple relations of cause and effect and going beyond single health determinants to understand the interrelationships between drivers of health outcomes. This is at odds with many public health programs and policies which focus on single (or a small number of) health issues or determinants or siloed programs that arise from outcome-specific priority-setting and funding mechanisms.

The combination of CLDs, to generate a CLD that represents the core issues that are common across communities provides powerful data to inform decision makers, (such as at state and regional levels of governance), about key determinants and assist in identifying high impact policy responses. An example of the importance of capturing this complexity is a school food study in New Zealand [23] which interviewed school lunch providers to identify potential intervention points to improve the programme and build a representative CLD. In this study twelve semi-structure interviews were analysed to create CLDs which were collated into one CLD which shows that providing healthy school lunches is driven by interactions between government nutrition policy, supply chain issues, ingredient supplies, school support and student demand [23]. This insight into what prima facie would appear a simple problem (how do we provide healthy school food) shows the importance of understanding this complexity to optimise potential interventions. While this example has a relatively narrow focus on food in schools in one district, the recent Lancet Commission on Obesity demonstrated how this complexity impacts planetary health on a global scale [24]. 

Notably the outcomes of any given GMB process are influenced significantly by the size and composition of the participant group, who act as the source of data that the GMB process aims to synthesise into a qualitative CLD [25]. An issue that is unique to a community may also be the most important driver for that community.

### Implications for practice

Currently, the quality of community-based prevention is a function of the capacity and capabilities of the prevention workforce, the breadth and strength of their partnerships and stakeholder networks, and their access to evidence-based prevention practices. There is a high rate of practitioner turnover, in an environment where capacity is difficult to build and sustain, and partnerships and networks are continually disrupted by organisational churn. In this context, there is a need for tools and practise that maximise the insights available to communities from systems-based approaches, especially where these approaches are embedded in larger initiatives that may be able to provide the framework for contextualisation and comparison. Merged maps could be used as a tool for communicating with policy makers to apply findings to a more general understanding of a complex problem providing new directions for the practice of prevention and population health more broadly. In another way, a merged map could be used as a ‘backbone’ or a starting point for future community GMBs to give them a head start in identifying harmful system drivers.

There are several tensions here, for example the move to system science reflects the acceptances that the complexity of the drivers of chronic disease are multiple and dynamic and engaging with this complexity is critical to a successful response. Conversely, capturing every variable and every relationship between these variables creates a ‘detail complexity’ that becomes overwhelming in the scale and breadth of the information presented. A second tension arises from the recognition that co-creation of chronic disease prevention is powerful because it engages communities to understand and address their unique characteristics that impact the success of generic health promotion interventions. The work presented here provides direction to a generalisable model of the causes of disease, which removes the heterogeneity between communities from the design of intervention. One potential solution is a distributed model of system thinking, whereby a health promotion workforce is trained in system thinking, able to use these techniques at a local level with local data, and share lessons back to a central repository towards a generalised model not just of cause and effect, as presented here, but also for tried and tested solutions operating across all facets of the systems as mapped.

### Future research

In Australia, as elsewhere in the world, government health prevention strategies are often in action or under development over a 3–4-year cycle. If the average time to translate research into practice is 17 years, [26] then research is currently of limited use to practice. Better methods are needed to translate and test lessons about prevention of chronic disease in a timely manner into standard practice, whether this is at a local, state, or national scale. The ability to co-create logic models is an important step towards a generic model of community wellbeing. Such models provide the potential for universal standards of practice so that multiple communities can understand the drivers of ill health in their context and access the best evidence and practice globally to optimise their response.

Sensitivity analysis around thresholds and the evaluation of the utility and/or acceptability of merged maps could be a potential focus for further research. Future work could also explore the possibility to iterate from merged maps over time to determine new community data to better understand whether this could improve them. Another research effort would be to begin with individual CLDs that had weighted edges i.e., the communities could nominate a value for the importance (or strength) of the connection. This would help to overcome the issue that all connections are treated equally, and only weighted by the number of communities that identified it.

### Strengths

A key strength of this merging approach is a simpler model that represents something that is more manageable, and generalisable than the complex original diagram, one which could be used as a valuable tool for advocacy and communication with community stakeholders.

The methods described here have the potential to be scaled up to represent even larger representations of key variables within CLDs examining complex problems.

## Limitations

As individual GMB processes can only be representative of the participants who contributed to the development of a given CLD, so too these results can only be representative of the constituent CLDs which were analysed to produce the final diagram. This diagram is not presented as a generic model of children and young people’s health and wellbeing cause in Victoria or generalisable beyond the 13 local governments included in this study. Rather, the diagram presented in this study demonstrates the utility of a process designed to centralise and share insights between communities using GMB and comparable participatory systems mapping processes in a shared problem space. Notably communities need to be relatively similar for this approach to synthesis to be meaningful.

During the variable name combination and CLD merging process subjective decisions were made which could not be entirely mitigated despite the inclusion of an independent review.

There is no established convention for determining a cut-point, ‘saturation’, or the minimum number of CLDs that are needed in order to be able to generate a useful merged version that adequately represents the core issues in a system. A possible way to address this would be to set a higher R + C value threshold for inclusion when merging a larger number of maps.

There is likely a relationship between the number of CLDs and the ‘right’ threshold to use, for example with a low number of CLDs important detail may be lost compared to merging with a greater number of CLDs (i.e., 100). If a greater number of CLDs are included a higher threshold could be reasonably set. Even so, some detail is lost in the merging process regardless of the number of CLDs included as some maps are more valuable than others. There is also a risk that an important variable only identified in one community is missed if the threshold of importance is > 1 in the identified community.

## Conclusion

The use of system science techniques to optimise efforts for health prevention is increasingly being used to inform government policy [11] and community-based prevention practice.

The opportunity to translate local contextually relevant data, combine common drivers and interrelationships across multiple communities. It is vital that researchers continue to tailor efforts to support these approaches in ways that advance the field globally.

## Data Availability

The datasets used and/or analysed during the current study are not publicly available. Reasonable data access request can be to the corresponding author via email s.ohalloran@deakin.edu.au.

## Abbreviations

GMB: Group model building
CLD: Causal loop diagrams
SNA: Social Network Analysis
DEMETEL: Decision-Making Trial and Evaluation Laboratory
VLGP: VicHealth Local Government Partnership
CtD: Connecting the Dots – creating solutions for lasting change
STICKE: Systems Thinking in Community Knowledge Exchange
